# PEDOT:PSS-MWCNT Nanocomposite Wire for Routing in Energy Harvesting Devices

**DOI:** 10.3390/mi16040382

**Published:** 2025-03-27

**Authors:** S. Haghgooye Shafagh, Imran Deen, Dhilippan Mamsapuram Panneerselvam, Muthukumaran Packirisamy

**Affiliations:** Optical-Bio Microsystems Laboratory, Micro-Nano-Bio Integration Center, Department of Mechanical, Industrial and Aerospace Engineering, Concordia University, Montreal, QC H3G 1M8, Canada; saber.haghgoo@yahoo.com (S.H.S.); imran.deen87@gmail.com (I.D.); dhilippan.mamsapurampanneerselvam@concordia.ca (D.M.P.)

**Keywords:** conductive polymer composites (CPCs), PEDOT:PSS, multiwalled carbon nanotubes (MWCNTs), energy harvesting

## Abstract

Polydimethylsiloxane (PDMS) and poly(3,4-ethylene dioxythiophene):poly(4-styrene-sulfonate) (PEDOT:PSS) composites were tested to determine their suitability for charging small-scale batteries in conjunction with a piezoelectric actuator as an energy harvester. Two different PEDOT:PSS patterns (zigzag and serpentine) were tested, and the maximum DC voltage of a system incorporating PEDOT:PSS was determined. The aim of this work is to study the effect of soft corners in the electrical routing of aircraft and IoT sensors. The zigzag and serpentine patterns were considered for this study because of their simplicity in design. Without the polymer, 2.3 V was produced by the actuator, while adding PEDOT:PSS resulted in the voltage being reduced to 1.7 V. The piezoelectric actuator was connected to a 3.6 V rechargeable Li-ion battery, and the battery’s voltage was recorded over 1 h. The voltage from the piezoelectric actuator was 3.8 V. Without PEDOT:PSS, the battery was charged to a maximum of 3 V. Adding the PEDOT:PSS to the circuit reduced the maximum charge to a voltage of 2 V. The results indicate that while PEDOT:PSS composites can be used in conjunction with piezoelectric energy harvesters, more work is still needed to optimize the system to increase efficiency and charging rates.

## 1. Introduction

With the advent of new manufacturing processes, flexible electronics have garnered much interest in the past few years. A flexible, easy-to-manufacture, and cost-effective device is a major challenge to producing the next generation of electronics. Processes such as 3D printing of liquid metals [1] and polymers [2] have made creating such devices feasible and cost-effective, and much research is being conducted on how flexible devices can be improved. Of particular interest are organic polymers, which are the main component of flexible electronics [3]. Creating an electrically conductive composite that can be used in a wide variety of applications is gaining much interest within the electronics industry [4,5]. One of the major challenges, however, remains finding a material that can be used as electrical routing while being stretched. Traditional wire-to-wire junctions break upon stretching, which can result in an increase in resistance of several ohms to gigaohms [6].

To this end, electrically conductive polymer composites (CPCs) are being investigated for a wide range of applications in the electronic and nanotechnology industries. They have unique features that allow them to be employed as smart materials in applications such as sensors [7,8,9,10], optoelectronics [11], and electromagnetic interference (EMI) shielding [12,13]. Polydimethylsiloxane (PDMS) [14,15,16] and poly(3,4-ethylene dioxythiophene):poly(4-styrene-sulfonate) (PEDOT:PSS) [9,17,18,19,20] are two different polymers that have been used to see the effect of the base polymer on conductivity. PDMS is a non-conductive polymer but more flexible; on the other hand, PEDOT:PSS is a highly conductive polymer but less flexible. PEDOT:PSS nanocomposites were found to have excellent flexibility and good adhesion to the different substrates. The polymers form a flexible matrix to which additives, such as multiwalled carbon nanotubes (MWCNTs) [16,21,22], are added to improve and tailor their material properties.

Furthermore, PEDOT:PSS has been shown to act as charge transport highways due to π–π interchain stacking [23,24]. PEDOT interaction with a dopant, such as graphene oxide [25] or MWCNTs [26], has been shown to improve conductivity. The addition of a plasticizer, such as PEG, helps reduce mass transport resistance, resulting in improved performance [27,28]. Additionally, adding organic solvents, such as isopropyl alcohol (IPA), methanol, or ethanol, has been shown to cause a separation of PEDOT and PSS, enabling the PEDOT to transition from a linear to a coiled or extended coil form; this allows for improved PEDOT interchaining and lowering of the energy barrier for charge transfers [29,30].

**Figure 1:**
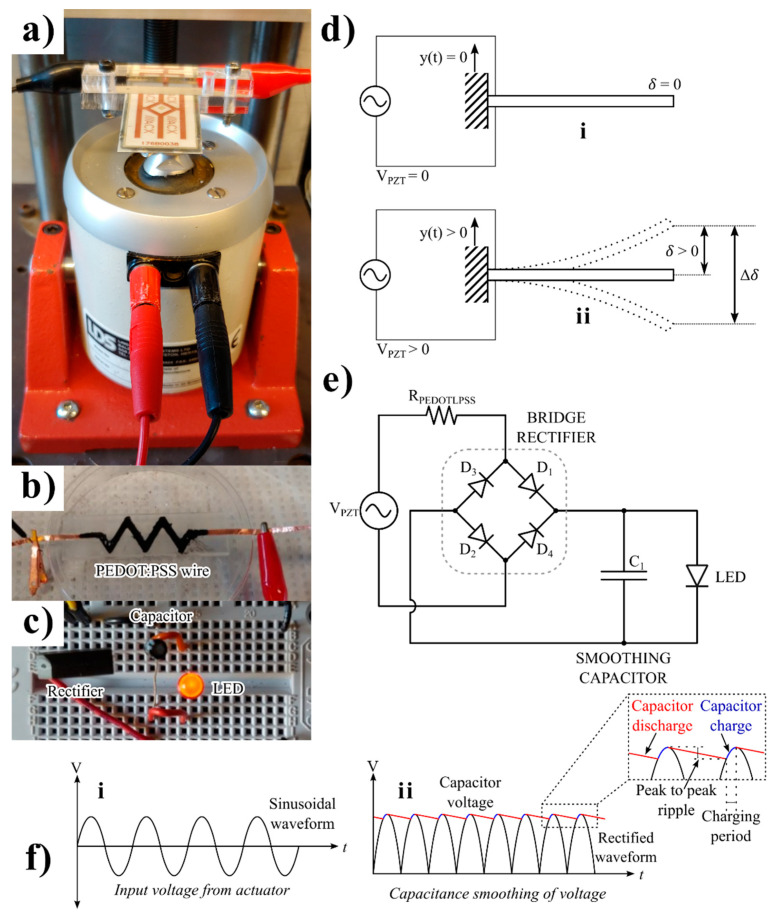
(**a**) A piezoelectric actuator hooked up to a shaker; (**b**) the PEDOT:PSS-MWCNT composite; (**c**) an LED lit up by energy produced from the actuator; (**d**) a diagram of a piezoelectric actuator in a cantilever beam set-up. The actuator is (**i**) at rest (y(t) = 0 and δ = 0) and produces no voltage (V_PZT_ = 0) and (**ii**) in motion (y(t) > 0 and δ > 0), producing an AC voltage (V_PZT_ > 0); (**e**) equivalent circuit of energy harvester; and (**f**) waveform of voltage from (**i**) a piezoelectric actuator and (**ii**) a full-bridge rectifier and the smoothing action of a reservoir capacitor.

The performance of modern wireless sensor networks and ultralow-power portable electronic devices depends on an unbounded amount of battery life. To harvest energy from environmental sources and create a sustainable auxiliary power source, extensive research has been carried out as part of the effort [31,32,33]. Energy harvesting is the process of gathering small amounts of energy from one or more nearby energy sources, storing them, and then using them when needed (see Figure 1). Sustainable energy harvesting is crucial for a consistent supply of electricity to the electronics in addition to the harvester’s output power [34,35,36,37].

A hybrid type of harvester on a single device was used, which harvests energy from one or more ambient energy sources (see Figure 1a–d) using a combination of different energy conversion mechanisms (see Figure 1e,f). These mechanisms included energy-transforming piezoelectric [38,39], thermoelectric [40,41], electrostatic [42], and triboelectric [43] phenomena. Piezoelectric materials can efficiently convert mechanical vibrational energy into electrical energy [44,45,46]. The authors in this work investigated the effect of length and different configurations (zigzag and serpentine) of PEDOT:SS under constant footprint in electrical routing from piezoelectric materials. The motivation of this work is to investigate any soft corners in the electrical routing of aircraft and IoT sensors. Thus, the authors selected the simplest design (serpentine and the zigzag) for their study. Numerous studies have been carried out to create cheap and effective vibration-based energy harvesting devices using piezoelectric materials, though the use of PDMS-PEDOT:PSS composites as routing wire has yet to be investigated.

## 2. Methodology

### 2.1. PEDOT:PSS-MWCNT Composites

In a glove box, MWCNTs were added to a solution of isopropyl alcohol (IPA) at 100:1, then ultrasonicated for 10 min using a VCX 500 ultrasonic processor (Sonics & Materials, Inc., Newtown, CT, USA). To this, PEDOT:PSS (Sigma-Aldrich, St. Louis, MO, USA) was added at 1:2 (by volume) and ultrasonicated for 2 min. One and four-tenths drops of glycerol and one drop of polyethylene glycol (PEG) (Sigma-Aldrich) were added for every milliliter of PEDOT:PSS (e.g., 10 mL PEDOT:PSS, 14 drops glycerol, 10 drops PEG).

**Figure 2:**
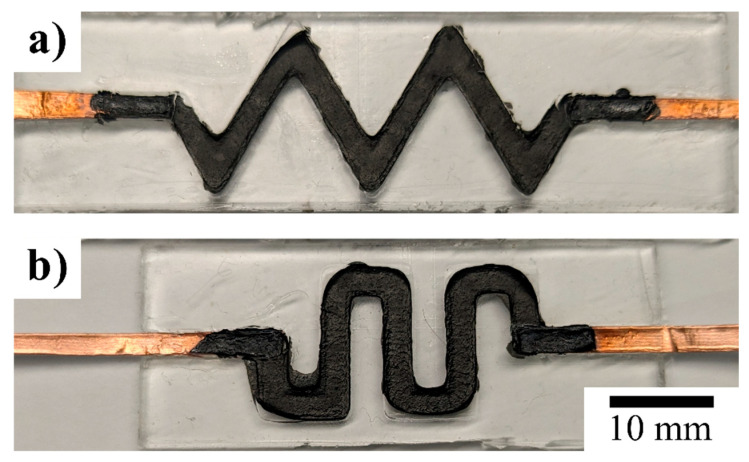
Top view of (**a**) serpentine and (**b**) zigzag channels filled with PEDOT:PSS and MWCNTs, with copper foils as electrodes.

The mixture was poured into the PDMS mold, and copper electrodes were placed so the tips were in contact with the mixture. The mixture was cured for 30 min at 70 °C or until the IPA evaporated. The mold was then sealed with a layer of PDMS and placed in the furnace at 70 °C for 15 min to cure (see Figure 2).

### 2.2. PDMS Molds

**Figure 3:**
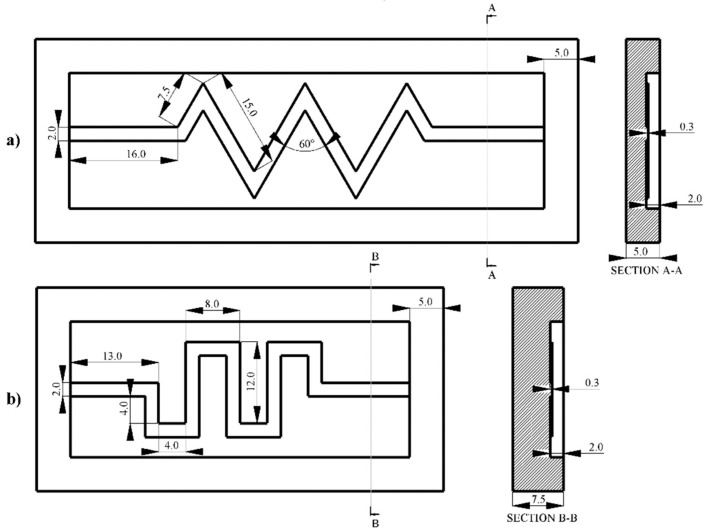
Dimensions of the mold for fabricating (**a**) zigzag and (**b**) serpentine PEDOT-MWCNT channels of 300 µm height with copper electrodes implanted (all dimensions in mm).

A PDMS (SYLGARD 184 Silicone Elastomer Kit, Dow Inc., Midland, MI, USA) mold was prepared by mixing the base elastomer and curing agent at 10:1, which was then placed in a desiccator hooked up to a Wob-l Dry Pump 2562B-01 (Welch, Niles, IL, USA) to remove air bubbles within the mixture. SolidWorks was used to design three different molds (see Figure 3), which were then made with a Form 2 Stereolithography (SLA) 3D Printer (Formlabs Co., Somerville, MA, USA). All channels were 2 mm wide and had a depth of 300 µm. The PDMS mixture was poured into a mold and placed in a furnace at 70 °C (Fisher Scientific, Pittsburgh, PA, USA) for 3 h to cure.

### 2.3. Experimental Set-Up

**Figure 4:**
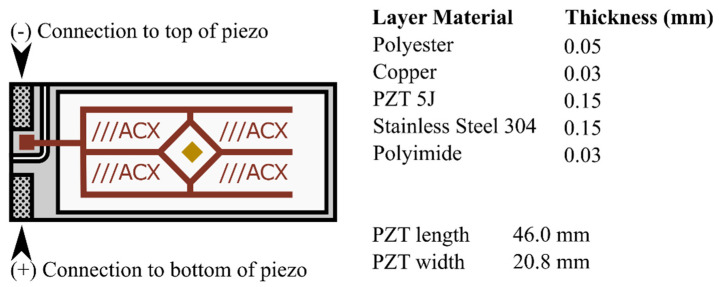
Lead–zirconium–titanate piezoelectric transducer.

A lead–zirconium–titanate S118-J1SS-1808YB Piezoelectric Bending Transducer (Mide Technology, Medford, MA, USA) was used to absorb mechanical energy and convert it to electricity (see Figure 4). The transducer was mounted to a Ling V203 Permanent Magnet Shaker (Ling Dynamic Systems Ltd., Hertfordshire, UK), and an Agilent 33220A 20 MHz Function/Arbitrary Waveform Generator (Agilent Technologies, Inc., Santa Clara, CA, USA) supplied the input voltage (*V_SUPPLY_*). Voltages ranging from 1 V to 10 V at frequencies ranging from 60 to 190 Hz were used as inputs to the shaker to measure the resonant frequency of the transducer. The d_33_ of the commercial transducer used was 485 m/V × 10^−12^.

**Figure 5:**
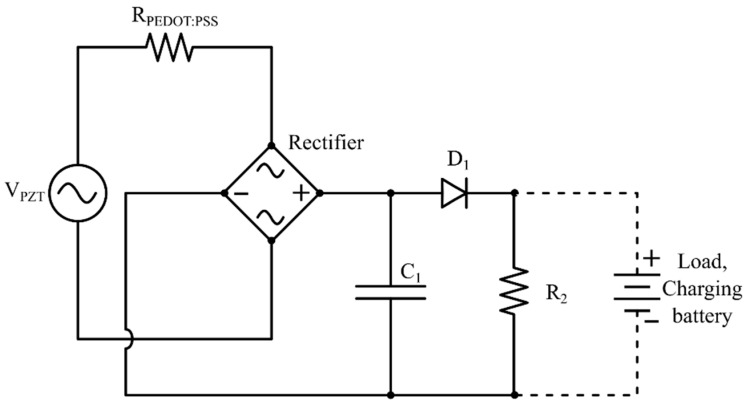
Equivalent circuit of energy harvesting set-up from piezoelectric transducer.

Figure 5 shows the schematic circuit of the set-up. *V_PZT_* is the voltage supplied by the piezoelectric actuator, *R*_1_ is the resistance from the PEDOT:PSS component (0 for control tests), *C*_1_ is the 10 µF smoothing capacitor, *D*_1_ is a 1N4001 diode, and *R*_2_ is a 30 k Ω resistor used for the time constant, τ, to equal 0.3 s. The output voltage from the piezoelectric transducer was converted from AC to DC through a 2KBP02M, 200 V 2A single-phase diode bridge rectifier. The rectified voltage was smoothed using *C*_1_, and *R*_1_ was added to the circuit to test the effect of PEDOT:PSS charging a commercial 3.6 V Li-ion rechargeable 2032 cell battery with a rating of 40 mAh.

### 2.4. Electrical Measurements

**Figure 6:**
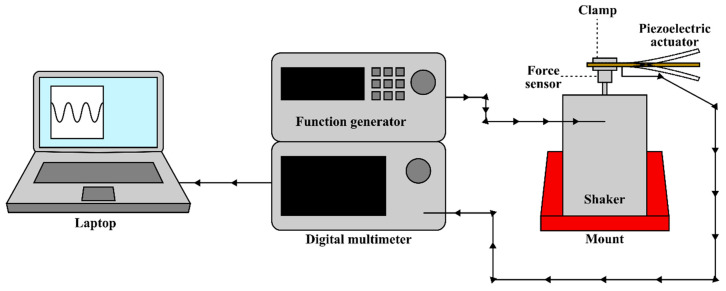
Schematic set-up for measuring current and voltage.

A Tektronix DPO2024B Digital Phosphor Oscilloscope (Tektronix Inc., Beaverton, OR, USA) was used to measure the voltage at different points in the circuit. The oscilloscope was connected to a computer using TekVISA v4.1.1, and the voltage waveform was recorded using Tektronix OpenChoice Desktop v2.6. Further electrical measurements were taken using a Keysight B2902A Precision Source Measurement Unit (Keysight, Santa Rosa, CA, USA), which was connected to a computer (Figure 6) and controlled using Quick I/V Measurement Software v4.2.2045.2760 (Keysight, USA).

**Figure 7:**
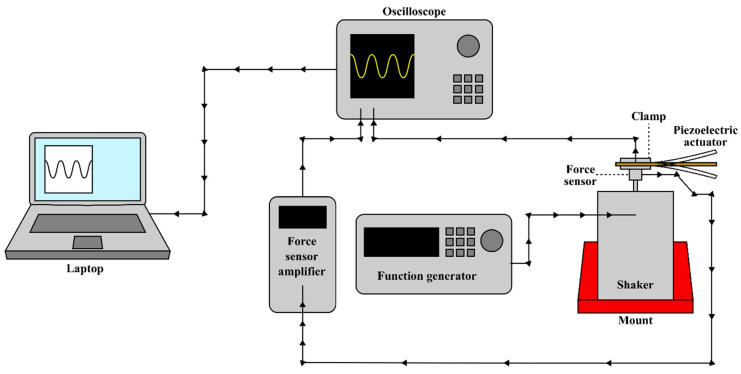
Schematic set-up for measuring force and voltage.

A 9712b50 force sensor (Kistler, Winterthur, Switzerland) connected to a Type 5010B charge amplifier (Kistler, Switzerland) was used to measure the force imparted by the shaker onto the piezoelectric actuator (see Figure 7).

#### Power and Efficiency Calculations

**Figure 8:**
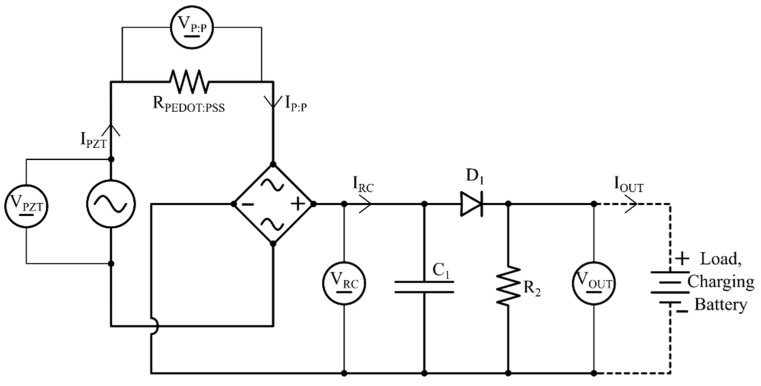
Equivalent circuit of set-up showing measured current and voltage for individual components.

Efficiency was calculated using Equation (1), where *η* is the efficiency, *P_IN_* is the input power calculated from the measured voltage and current, *P* = *IV*, and *P_OUT_* is the output power provided by individual stages (see Figure 8). The input and output powers of each cascaded block are interlinked via a series of components, and the components’ performance regarding any stray capacitance and internal resistance are encountered as lumped parameters by this modeling. The power produced by the piezoelectric actuator (*P_PZT_*), the power flow in the PEDOT:PSS component (*P_P:P_*), the rectifier (*P_RC_*), and the RC network (*P_OUT_*) were measured.

(1) $$\eta =\frac{P_{OUT}}{P_{IN}}$$

**Figure 9:**
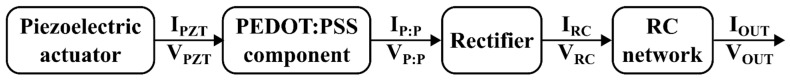
Efficiency between components.

By measuring the voltage and current for the piezoelectric actuator (*V_PZT_*, *I_PZT_*), the PEDOT:PSS component (*V_P:P_*, *I_P:P_*), the rectifier (*V_RC_*, *I_RC_*), and the RC network (*V_OUT_*, *I_OUT_*) (see Figure 9), the efficiency of each component can be calculated using Equations (2)–(4). The total efficiency of the circuit (*η_T_*) was calculated using Equation (5).

(2) $$\eta_{P:P}=\frac{P_{P:P}}{P_{PZT}}=\frac{V_{P:P}I_{P:P}}{V_{PZT}I_{PZT}}$$

(3) $$\eta_{RC}=\frac{P_{RC}}{P_{P:P}}=\frac{V_{RC}I_{RC}}{V_{P:P}I_{P:P}}$$

(4) $$\eta_{OUT}=\frac{P_{OUT}}{P_{RC}}=\frac{V_{OUT}I_{OUT}}{V_{RC}I_{RC}}$$

(5) $$\eta_{T}=\eta_{P:P}\times \eta_{RC}\times \eta_{OUT}$$

## 3. Results and Discussion

**Figure 10:**
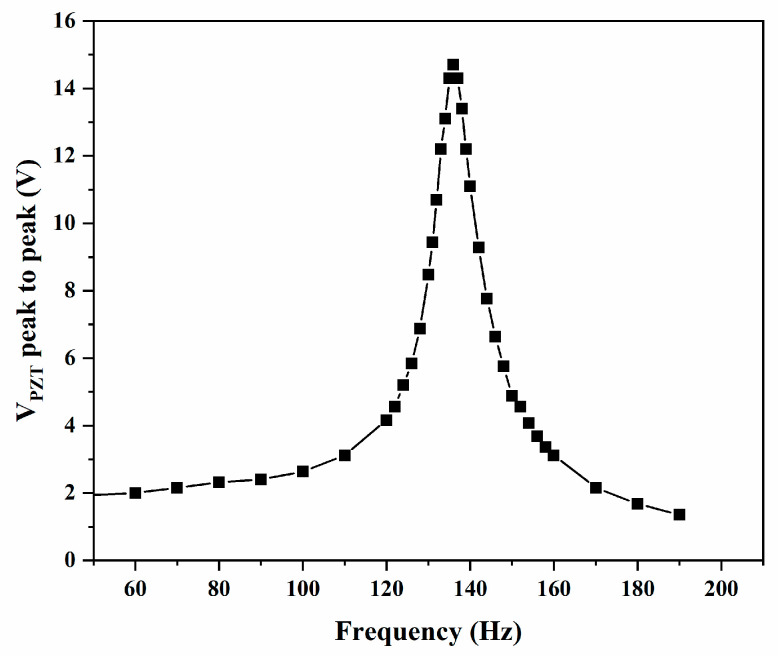
Maximum voltage of piezoelectric transducer, from 60 to 190 Hz.

The resonant frequency (*f_RES_*) was experimentally determined by applying 10 V to the shaker from 60 to 190 Hz. The maximum voltage at each frequency, and the results indicate that *f_RES_* can be found at 135 Hz (see Figure 10). Applying a potential at 135 Hz through the shaker will thus produce the maximum voltage from the piezoelectric transducer.

**Figure 11:**
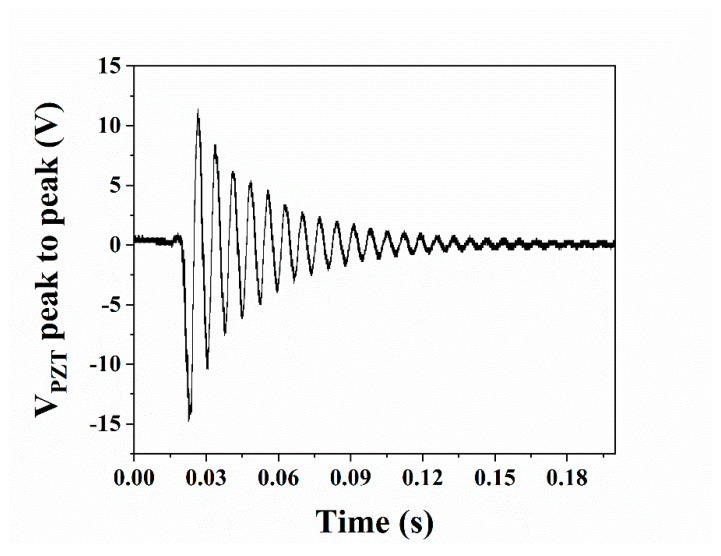
Voltage produced from piezoelectric actuator during “flick test”.

A “flick test” was conducted where the piezoelectric actuator was physically oscillated, and the voltage produced was recorded. Figure 11 shows *V_PZT_*, a typical dampened waveform pattern with a period, T, of 0.073 s. Taking *f =* 1/T, the resonant frequency is 136 Hz.

**Figure 12:**
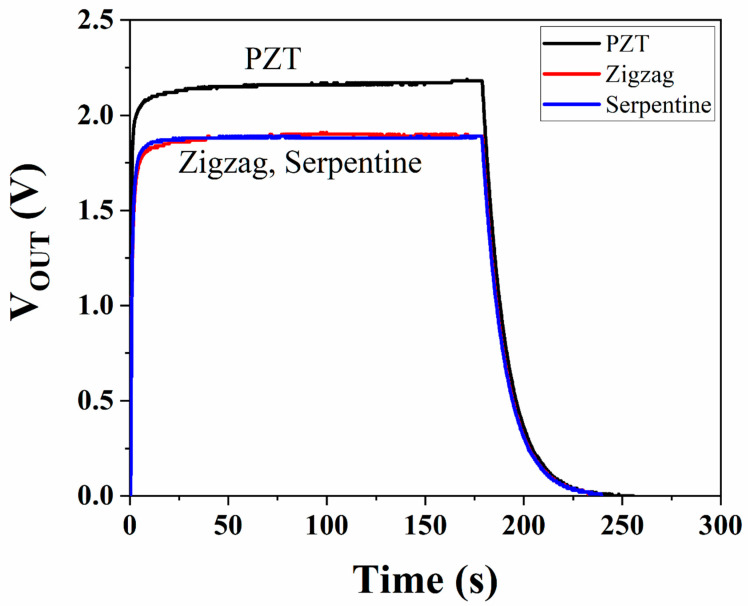
Maximum DC voltage after smoothing recorded from piezoelectric transducer and from PEDOT:PSS components.

The voltage was measured after having been rectified and smoothed, and the effect of using PEDOT:PSS in the circuit was measured (see Figure 12). The PZT voltage (when there was no extra resistance from the PEDOT:PSS components in the circuit) is approximately 2.3 V, while adding a PEDOT:PSS component, regardless of the channel type, results in a voltage reduction to obtain 1.7 V.

**Figure 13:**
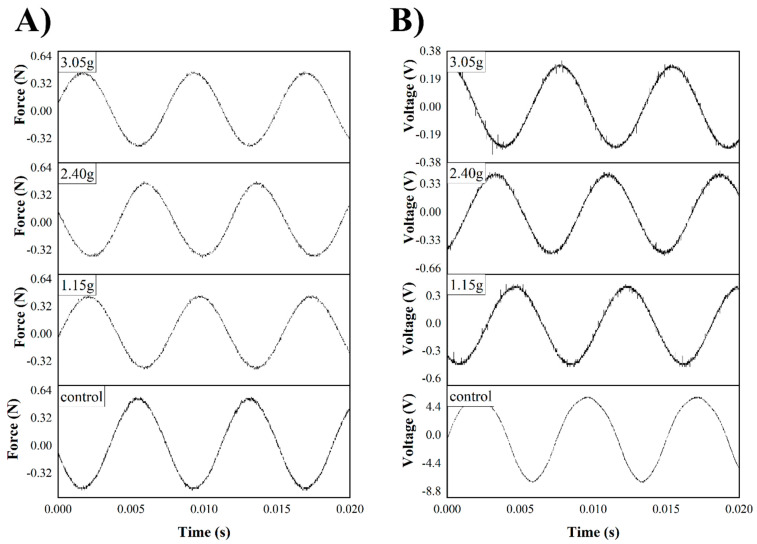
(**A**) Shaker force and (**B**) voltage from actuator before and after dampening with 1.15, 2.40, and 3.05 g loads.

**Figure 14:**
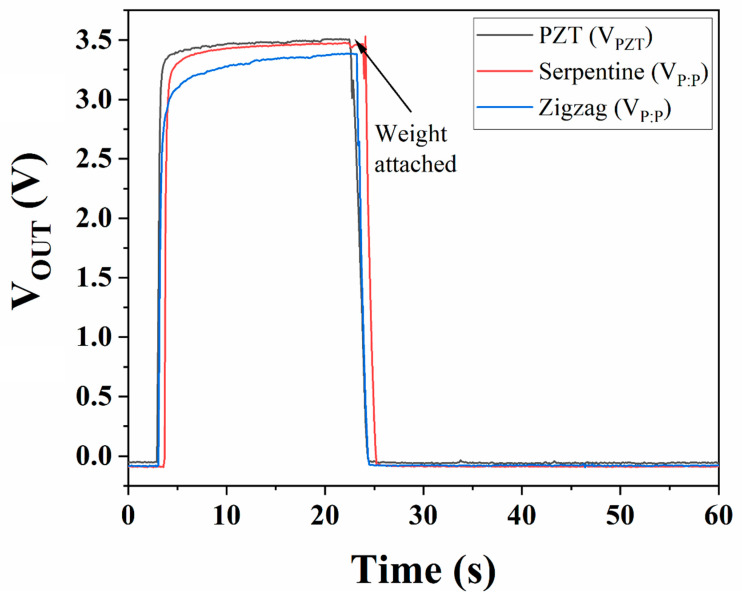
DC voltage before and after dampening with a 1.15 g load, recorded from the piezoelectric transducer and from PEDOT:PSS components.

In other tests, small weights (binder clips) were added and clipped to the tip of the piezoelectric energy harvester to see their effect on the output voltage as well as force. Weights of 1.15 g, 2.40 g, and 3.05 g were added. According to the results (see Figure 13), the force imparted on the actuator drops from 0.57 N to 0.48 N when a load is added to the cantilever, but the voltage decreases to 300 mV from 5.8 V, showing that damping to the piezoelectric actuator will cause a decrease in voltage by 95%. Figure 14 also shows that adding weight to the tip of the energy harvester causes an immediate drop in the smoothed voltage to nearly 0 V as well, regardless of whether PEDOT:PSS is present.

**Table 1:** Maximum recorded force and voltage after weight added to piezoelectric actuator.

	0 g	1.15 g	2.40 g	3.05 g
**Shaker force (N)**	0.57	0.48	0.46	0.46
**Voltage, V_PZT_ (V)**	6.00	0.49	0.45	0.32

The efficiency, *η*, was calculated by recording the current and voltage at different junctions within the circuit and calculating the power. Efficiency was calculated in three different circuits, with and without the PEDOT:PSS component, to see the effect of implementing conductive PEDOT:PSS on efficiency.

Table 2 shows the power calculation using *P* = *IV*. Table 3 and Table 4 show the power when the PEDOT:PSS components have been inserted into the circuit. The circuit loses about 50% of its power in the bridge rectifier and another 50% in the RC time constant. Based on these results, using PEDOT:PSS in the circuit does not have a significant effect on efficiency (see Table 5), though the performance of the circuit can be improved by incorporating low-power diodes in place of the bridge rectifier.

**Figure 15:**
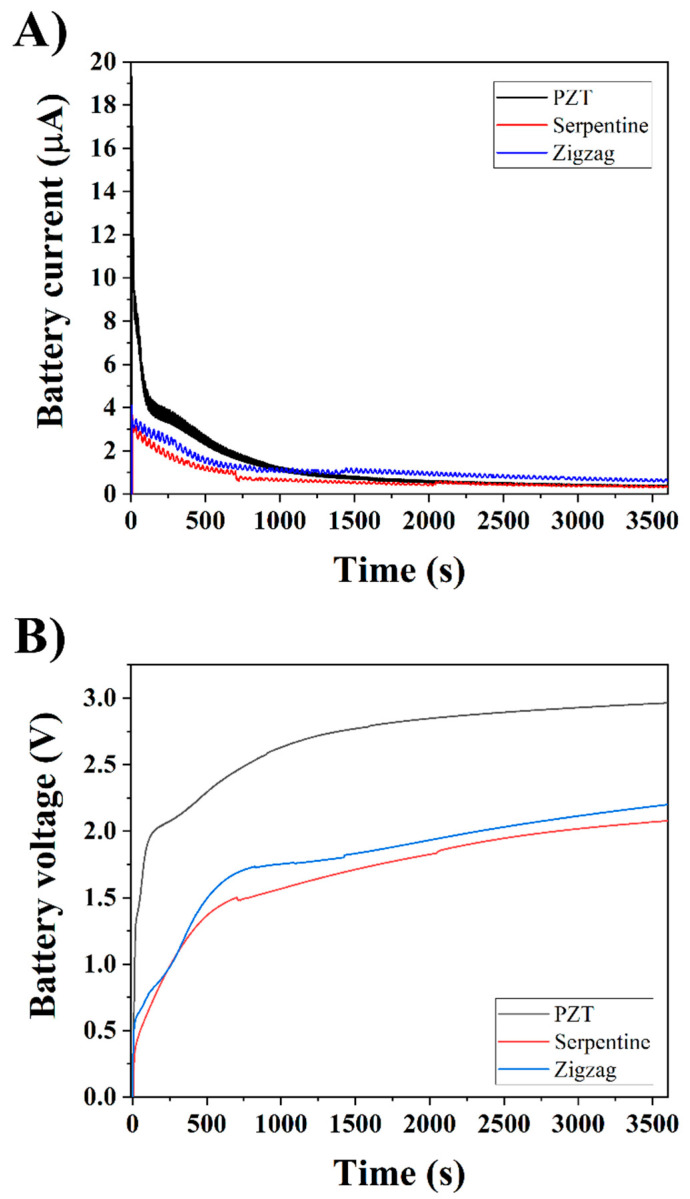
Real-time (**A**) current and (**B**) voltage recorded from the battery during charging from the piezoelectric transducer and from PEDOT:PSS components.

In the last step, a 3.6 V Li-ion rechargeable 2032 cell battery was tested to determine the effect of PEDOT:PSS on charging. Figure 15 shows the charging rate of the battery with and without PEDOT:PSS. Two different patterns (zigzag and serpentine) were tested, and the voltage was recorded over 1 h, and the voltage from the piezoelectric actuator was 3.8 V. Without PEDOT:PSS, the battery was charged to a maximum of 3 V. By adding the PEDOT:PSS to the circuit, the maximum charge was reduced. The results show the battery will be charged to a maximum of 2 V after 1 h, for both serpentine and zigzag patterns.

Owing to these works, the authors could study the effect of different configurations of the conductive polymer. The successful validation to charge a bare rechargeable lithium battery, with and without conductive polymer, follows the principle that these routing mechanisms are adequate for electrical charge conduction of piezoelectricity. The effect of this work can be highlighted in aircraft, where electrical routing of the vibrations via a conductive polymer can function as a miniature powerhouse to entrap all the vibration as electrical energy. The harvested (routed and stored) electricity can help to power soft IoT sensors for commercial applications. The authors noticed minimal efficiency variations (0.04%) between the selected zigzag and serpentine designs. However, the authors noticed a significant 33% decline in efficiency while charging the bare li-ion battery. It should be noted here that the authors used commercially available electronic components to design these experiments. With proper electronic design and device optimization, these efficiency losses can be abruptly mitigated.

## 4. Conclusions

These results show that it is possible to use a PEDOT:PSS in conjunction with a piezoelectric actuator to charge small components, such as a rechargeable 3.6 V Li-ion battery. Using PEDOT:PSS for charging a battery is feasible; however, it reduced the charging by around 33%. The efficiency test showed that using conductive PEDOT:PSS does not have a notable impact on total efficiency. It was found that any damping would prevent charging as it massively decreases the voltage the actuator produces. To the best of our knowledge, using a conductive polymer to charge the batteries has not been previously investigated and is a promising technology for future electrical routing research.

For future research, adding different metal particles, such as gold, can be investigated. A more effective approach to increase the efficiency of the conductive polymer is possible, though it might reduce the flexibility of the sample.

## Figures and Tables

**Table 2:** Measured RMS voltage and current without any PEDOT:PSS component.

	RMS Voltage (V)	RMS Current (A)	Power (W)
**PZT, V_PZT_**	5.94	4.90 × 10^−4^	2.91 × 10^−3^
**Rectifier, V_RC_**	5.52	2.50 × 10^−4^	1.38 × 10^−3^
**RC network, V_out_**	3.89	1.80 × 10^−4^	0.70 × 10^−3^

**Table 3:** Measured RMS voltage and current with a zigzag PEDOT:PSS component included.

	RMS Voltage (V)	RMS Current (A)	Power (W)
**PZT, V_PZT_**	5.94	4.90 × 10^−4^	3.16 × 10^−3^
**PEDOT:PSS, V_P:P_**	5.52	4.60 × 10^−4^	2.92 × 10^−3^
**Rectifier, V_RC_**	5.86	2.50 × 10^−4^	1.47 × 10^−3^
**RC network, V_out_**	4.12	1.80 × 10^−4^	0.74 × 10^−3^

**Table 4:** Measured RMS voltage and current with a serpentine PEDOT:PSS component included.

	RMS Voltage (V)	RMS Current (A)	Power (W)
**PZT, V_PZT_**	6.55	5.10 × 10^−4^	3.34 × 10^−3^
**PEDOT:PSS, V_P:P_**	6.53	4.90 × 10^−4^	3.20 × 10^−3^
**Rectifier, V_RC_**	5.91	2.50 × 10^−4^	1.48 × 10^−3^
**RC network, V_out_**	4.20	1.80 × 10^−4^	0.76 × 10^−3^

**Table 5:** Cascading efficiency between components.

	Efficiency of Control	Efficiency with Zigzag Line	Efficiency with Serpentine Line
**PZT to PEDOT:PSS**	n/a	0.92	0.96
**PEDOT:PSS to rectifier**	0.47 *	0.50	0.46
**Rectifier to RC network**	0.51	0.51	0.51
**Total efficiency**	0.24	0.23	0.23

(* efficiency for PZT to rectifier).

## Data Availability

Data available on request from the correspondence author.
